# Chronic venous insufficiency increases postoperative complications after Mohs micrographic surgery for lower extremity nonmelanoma skin cancer: A propensity-matched retrospective cohort study

**DOI:** 10.1016/j.jdin.2025.09.008

**Published:** 2025-10-03

**Authors:** Sahithi Talasila, Umer Nadir, George M. Jeha, Stanislav N. Tolkachjov

**Affiliations:** a Transitional Year Residency, Orange Park Hospital, Orange Park, Florida; b Epiphany Dermatology, Dallas, Texas; c Division of Dermatology, Baylor University Medical Center, Dallas, Texas; d Texas A&M College of Medicine, Dallas, Texas; e Department of Dermatology, The University of Texas at Southwestern Medical Center, Dallas, Texas

**Keywords:** basal cell carcinoma, chronic venous insufficiency, Mohs micrographic surgery, nonmelanoma skin cancer, postoperative outcomes, squamous cell carcinoma

*To the Editor:* Chronic venous insufficiency (CVI) is characterized by impaired venous return, resulting in lower extremity (LE) edema, hypoperfusion, and a proinflammatory microenvironment.^1^ Prior research, notably in orthopedic surgery literature, has shown these pathophysiological changes increase susceptibility to post-surgical complications and infections.^2^ Mohs micrographic surgery is indicated for nonmelanoma skin cancers of the LE that are recurrent, have aggressive histologic features (eg, morpheaform, infiltrative, and perineural), or involve the nail unit, ankle, or pretibial surface.^3^ Yet, large-scale data evaluating the impact of CVI on dermatologic surgical outcomes remain limited.

In this retrospective cohort study using the TriNetX Research Network between July 12, 2020 and July 12, 2025, we evaluated whether CVI is associated with increased postoperative complication rates in patients undergoing MMS for LE nonmelanoma skin cancer (NMSC). Patients with CVI were identified with the International Classification of Diseases, 10th revision code for CVI (I87.2) [ICD-10-CM].^4^ We excluded patients with disorders known to impair wound healing (epidermolysis bullosa, systemic sclerosis, Ehlers-Danlos, Marfan syndrome), any systemic infection documented on the day of surgery, or a history of postoperative complications prior to the study period to minimize confounding from patients predisposed to poor outcomes. Patients with CVI were propensity score matched on age, demographics, immunosuppressive medications, and co-morbid conditions, including diabetes mellitus, to a comparator cohort without CVI (Table I). Postoperative outcomes were assessed at 30-days and 90-days after surgery with risk ratios (RR).

**Table I:** Baseline demographic characteristics of the chronic venous insufficiency (CVI) and no CVI cohorts before and after propensity score matching (PSM)

	Baseline characteristics before PSM			Baseline characteristics after PSM		
	CVI cohort [*n* (%)]∗ (*n* = 3182)	No CVI cohort [*n* (%)] (*n* = 25690)	*P* value†	CVI cohort [*n* (%)] (*n* = 3169)	No CVI cohort [*n* (%)] (*n* = 3169)	*P* value
Age at index, mean (SD)	77.7 (8.8)	72.5 (10.2)	<.001	77.6 (8.8)	77.5 (8.8)	.69
Male	1448 (45.5%)	11894 (46.3%)	.014	1444 (45.6%)	1441 (45.5%)	.94
Female	1718 (54.0%)	13691 (53.3%)	.016	1709 (53.9%)	1710 (54.0%)	.98
White	3052 (95.9%)	24282 (94.5%)	.001	3040 (95.9%)	3055 (96.4%)	.33
Black or African American	10 (0.3%)	47 (0.2%)	.12	10 (0.3%)	10 (0.3%)	1
Asian	10 (0.3%)	31 (0.1%)	.006	10 (0.3%)	10 (0.3%)	1
Hispanic or Latino	15 (0.5%)	96 (0.4%)	.4	13 (0.4%)	18 (0.6%)	.37
Not Hispanic or Latino	2594 (81.5%)	19954 (77.7%)	<.001	2585 (81.6%)	2579 (81.4%)	.85
Overweight and obesity	454 (14.3%)	1460 (5.7%)	<.001	446 (14.1%)	460 (14.5%)	.62
Diabetes mellitus	594 (18.7%)	2391 (9.3%)	<.001	586 (18.5%)	587 (18.5%)	.97
Smoking status	97 (3.0%)	672 (2.6%)	.15	96 (3.0%)	91 (2.9%)	.71
Peripheral arterial disease	90 (2.8%)	144 (0.6%)	<.001	82 (2.6%)	73 (2.3%)	.46
Tumor necrosis factor alpha (TNF-α) inhibitor use	31 (1.0%)	169 (0.7%)	.042	31 (1.0%)	28 (0.9%)	.7
Mycophenolate mofetil use	35 (1.1%)	274 (1.1%)	.86	35 (1.1%)	20 (0.6%)	.042
Methotrexate use	54 (1.7%)	244 (0.9%)	<.001	54 (1.7%)	53 (1.7%)	.92
Tacrolimus use	89 (2.8%)	596 (2.3%)	.095	89 (2.8%)	84 (2.7%)	.7
Cyclosporine use	40 (1.3%)	236 (0.9%)	.064	39 (1.2%)	29 (0.9%)	.22
Systemic corticosteroid use	1605 (xs50.4%)	8117 (31.6%)	<.001	1592 (50.2%)	1601 (50.5%)	.82

*SD*, Standard deviation.

∗ Percentages will not add up to 100% as certain patients did not have all demographics denoted comprehensively in their medical records.

† A two-sided *P* value <.05 was considered statistically significant.

We identified 3169 patients with CVI and 3169 matched controls (Table II). At 30 days postoperatively, CVI was significantly associated with increased risk of infection (RR 2.16, *P* = .007), non-healing wounds (RR 3.70, *P* < .001), and more debridement procedures (RR 4.69, *P* < .001). At 90 days, CVI remained significantly associated with increased risk of infection (RR 1.85, *P* = .009), non-healing wounds (RR 2.72, *P* < .001), more debridement procedures (RR 4.18, *P* < .001), and wound dehiscence (RR 2.46, *P* = .003). Emergency department visits and inflammatory markers (WBC, CRP, ESR) did not differ between cohorts, though albumin levels were lower in CVI patients (*P* = .004).

**Table II:** Risk of postoperative complications 30 days and 90 days following Mohs micrographic surgery for nonmelanoma skin cancer in patients diagnosed with chronic venous insufficiency

Outcomes	CVI cohort	No CVI cohort	Risk ratio (95% CI)	*P* value	CVI cohort	No CVI cohort	Risk ratio (95% CI)	*P* value
	Number of eligible individuals∗ (no. of outcomes, 30-d)†	Number of eligible individuals (no. of outcomes, 30-d)			Number of eligible individuals (no. of outcomes, 90-d)‡	Number of eligible individuals (no. of outcomes, 90-d)		
Postoperative infection	2995 (36)	3058 (17)	2.16 (1.22, 3.84)	.007	2995 (49)	3058 (27)	1.85 (1.16, 2.96)	.009
Delayed wound healing	3000 (36)	3085 (10)	3.70 (1.84, 7.45)	<.001	3000 (66)	3085 (25)	2.72 (1.72, 4.29)	<.001
Dehiscence	3064 (17)	3106 (10)	1.72 (0.79, 3.76)	.17	3064 (34)	3106 (14)	2.46 (1.32, 4.58)	.003
Debridement	2967 (45)	3093 (10)	4.69 (2.37, 9.29)	<.001	2967 (72)	3093 (18)	4.18 (2.49, 6.97)	<.001
Emergency department visits	1723 (10)	2001 (10)	1.16 (0.49, 2.78)	.74	1723 (28)	2001 (26)	1.25 (0.74, 2.13)	.41

	Mean (SD)	Mean (SD)	*P* value	Mean (SD)	Mean (SD)	*P* value§
White blood cell count (10ˆ3/uL)	7.41 (4.173)	22.77 (268.34)	.28	7.28 (3.58)	7.10 (3.37)	.33
C-Reactive protein (mg/L)	29.49 (45.16)	22.28 (31.12)	.53	22.21 (43.45)	26.68 (49.68)	.56
Erythrocyte sedimentation rate (mm/h)	28.15 (26.57)	23.19 (20.57)	.46	27.37 (24.19)	21.95 (18.93)	.17
Albumin (g/dL)	3.94 (0.46)	4.04 (0.42)	.004	3.97 (0.45)	4.04 (0.44)	.004

*10ˆ3/uL*, Thousands of cells per microliter; *CI*, confidence interval; *CVI*, chronic venous insufficiency; *g/dL*, grams per deciliter; *mg/L*, milligrams per liter; *mm/h*, millimeters per hour; *SD*, standard deviation.

∗ The counts in the “Number of eligible individuals” for each postoperative outcome differ slightly because each analysis was conducted independently. For each specific complication (eg, infection, dehiscence, and delayed healing), only patients with that complication documented prior to the index surgery were excluded. As a result, the denominators vary slightly across outcomes.

† No. of outcomes, 30-d: Number of outcomes within 30 d following the index Mohs micrographic surgery.

‡ No. of outcomes, 90-d: Number of outcomes within 90 d following the index Mohs micrographic surgery.

§ A two-sided *P* value <.05 was considered statistically significant.

This study provides evidence supporting an increased risk of postoperative complications, including infection, delayed wound healing, more debridement procedures, and dehiscence in CVI patients undergoing MMS for LE NMSC. Limitations include the use of ICD-10-CM coding and potential for unknown confounding variables. The dataset lacked clinical details on recurrence, histologic subtype, and specific LE location, preventing assessment of whether MMS cases in our study met guideline-supported indications.^3^ It also lacked information on repair type, as procedure codes for closure techniques are inconsistently applied and lack linkage to anatomical site, limiting the ability to distinguish between primary closures and flap repairs when interpreting wound dehiscence outcomes. Because repair type influences complication risk–for example, prior research has shown primary bilayered closures carry lower infection and bleeding risks than secondary intention healing–future studies should evaluate these details.^5^ Future research may also evaluate prospective data and causal factors associated with post-surgical complications. Despite limitations, our manuscript describes an epidemiological conclusion that patients with CVI have poorer outcomes, and dermatologic surgeons should be ready to prevent and address these complications.

## Conflicts of interest

Dr Tolkachjov is an investigator/speaker for Bioventus, Kerecis, Boehringer Ingelheim, Regeneron, and CASTLE. Drs Talasila, Nadir, and Jeha have no conflicts of interest to declare.
